# Supplementary material for: Differences in GenBank and RefSeq annotations may affect genomics data interpretation for Pseudomonas putida KT2440
Source: mSphere. 2025 Oct 2;10(10):e00391-25. doi: 10.1128/msphere.00391-25 (PMC12570471; doi:10.1128/msphere.00391-25)
Supplement: Supplemental figures and tables — Figures S1 to S3; Tables S1 and S2. [file msphere.00391-25-s0004.pdf]

Differences in GenBank and RefSeq annotations may affect genomics data interpretation for *Pseudomonas putida* KT2440

Guilherme Marcelino Viana de Siqueira[1,2,3], Thomas Eng[2,3], Aindrila Mukhopadhyay[2,3,4], and María-Eugenia Guazzaroni[1#]

[1] Department of Biology, Faculty of Philosophy, Sciences and Letters at Ribeirão Preto, University of São Paulo, Ribeirão Preto, SP, 14040-900, Brazil

[2] The Joint BioEnergy Institute, Lawrence Berkeley National Laboratory, Emeryville, CA, 94608, USA

[3] Biological Systems and Engineering Division, Lawrence Berkeley National Laboratory, Berkeley, CA, 94720, USA

[4] Environmental Genomics and Systems Biology Division, Lawrence Berkeley National Laboratory, Berkeley, CA, 94720, USA

# correspondence: meguazzaroni@ffclrp.usp.br

SUPPLEMENTAL INFORMATION

Tables

Table S1. Summary information on the RNAseq datasets used in this work.

Table S2. Comparison of differences in ORF sizes for shifted genes between GenBank and RefSeq annotations.

Figures

Figure S1. Reference genome annotation usage in ALEdb *P. putida* KT2440 mutation records.

Figure S2. Overrepresentation analysis for the cluster of orthologous genes (COG) categories of shifted genes annotated with “Cellular processes and signaling” functions.

Figure S3. Differences in the output of RNAseq analyses due to the use of different reference transcriptomes.

Table S1. Summary information on the RNAseq datasets used in this work.

BioProject Acc.	Base Culture Media	Growth Condition(s)	Publication (Referenced in Main Text)	Reference Transcriptome in Original Publication
PRJNA480068	minimal salt medium	Myristic acid	Not published	-
PRJNA796354	minimal salt medium	Various carbon sources	(19)	AE015451.2 (GenBank)
PRJNA885112	LB	Selenium	(27)	GCA_000014625.1 (*P. aeruginosa* GenBank acc.)
PRJNA630234	minimal salt medium	Ionic liquids	(28)	NC_002947.4 (RefSeq)
PRJNA533248	minimal salt medium	Nutrient-limited bioreactor	(29)	NC_002947.4 (RefSeq)
PRJNA486005	minimal salt medium	Various carbon sources	(30)	NC_002947.3 (RefSeq)
PRJNA341451	minimal salt medium	Solvents (toluene)	(31)	NC_002947 (RefSeq)
PRJNA338603	minimal salt medium	Osmotic and oxidative stresses, antibiotics	(32)	NC_002947.3 (RefSeq)
PRJNA450701	minimal salt medium	Zinc	(33)	PseudomonasDB (Derived from GenBank)

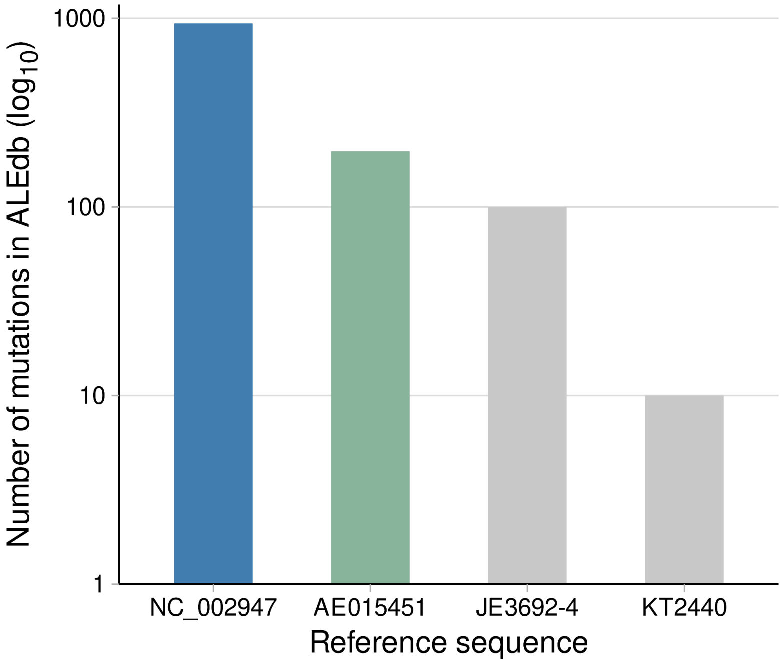

Figure S1. Reference genome annotation usage in ALEdb *P. putida* KT2440 mutation records. Data from ALEdb (https://aledb.org/) was downloaded using *P. putida*'s KT2440 taxonomic ID (160488) as the single search term in December 2024. Of note, there are 3 mutations in this database (at genomic positions 1,201,211; 3,702,766; and 4,993,315) that fall in the “grey zone” between annotations. This means that if the GenBank annotation of the genome is considered, the first two mutations are within ORFs (namely PP_1051, which encodes a Type II secretion system protein, and PP_3269, which encodes a general stress protein). Alternatively, if the RefSeq annotation is considered, only the third one is within the span of an ORF (PP_4402, which encodes the beta subunit of an alpha-ketoacid dehydrogenase).

Table S2. Comparison of differences in ORF sizes for shifted genes between GenBank and RefSeq annotations.

Number of affected loci	Affected extremity	Min difference (bp)	Max difference (bp)	Difference median (bp)	Outcome on relative ORF size
75	3'	3	114	31.5	RefSeq is larger
380	3'	3	261	33	GenBank is larger
78	5'	3	353	27	RefSeq is larger
364	5'	3	297	30	GenBank is larger

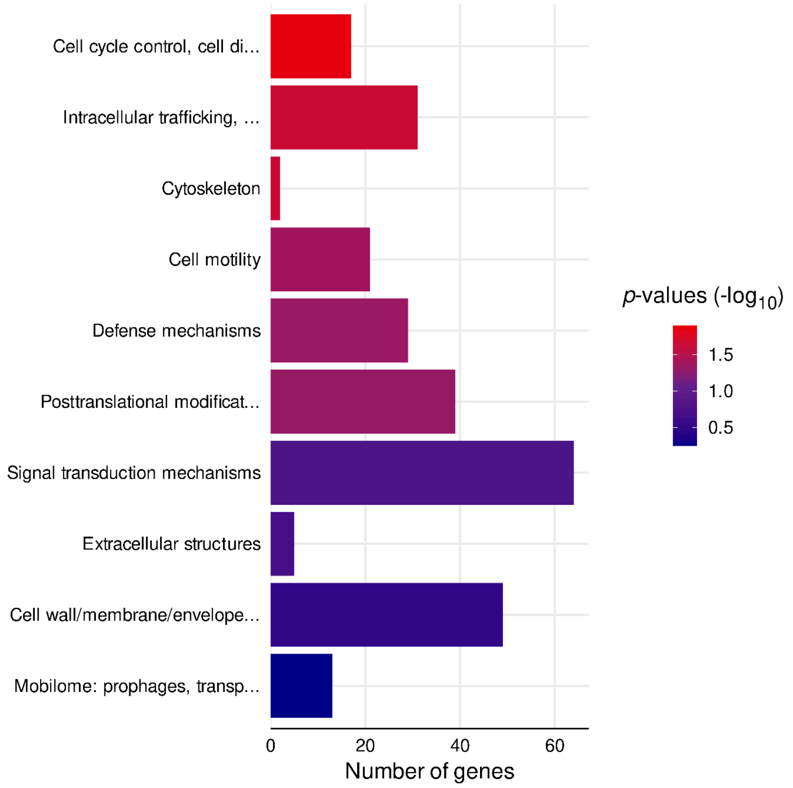

Figure S2. Overrepresentation analysis for the cluster of orthologous genes (COG) categories of shifted genes annotated with "Cellular processes and signaling" functions. In this figure, the number of shifted genes within each of the ten COG categories in this group is represented by horizontal bars. The color scale indicates *p*-values from one-tailed Fisher's exact tests, which assess the association between shifted and unshifted genes for each specific annotation relative to the rest of the genes with COG annotations.

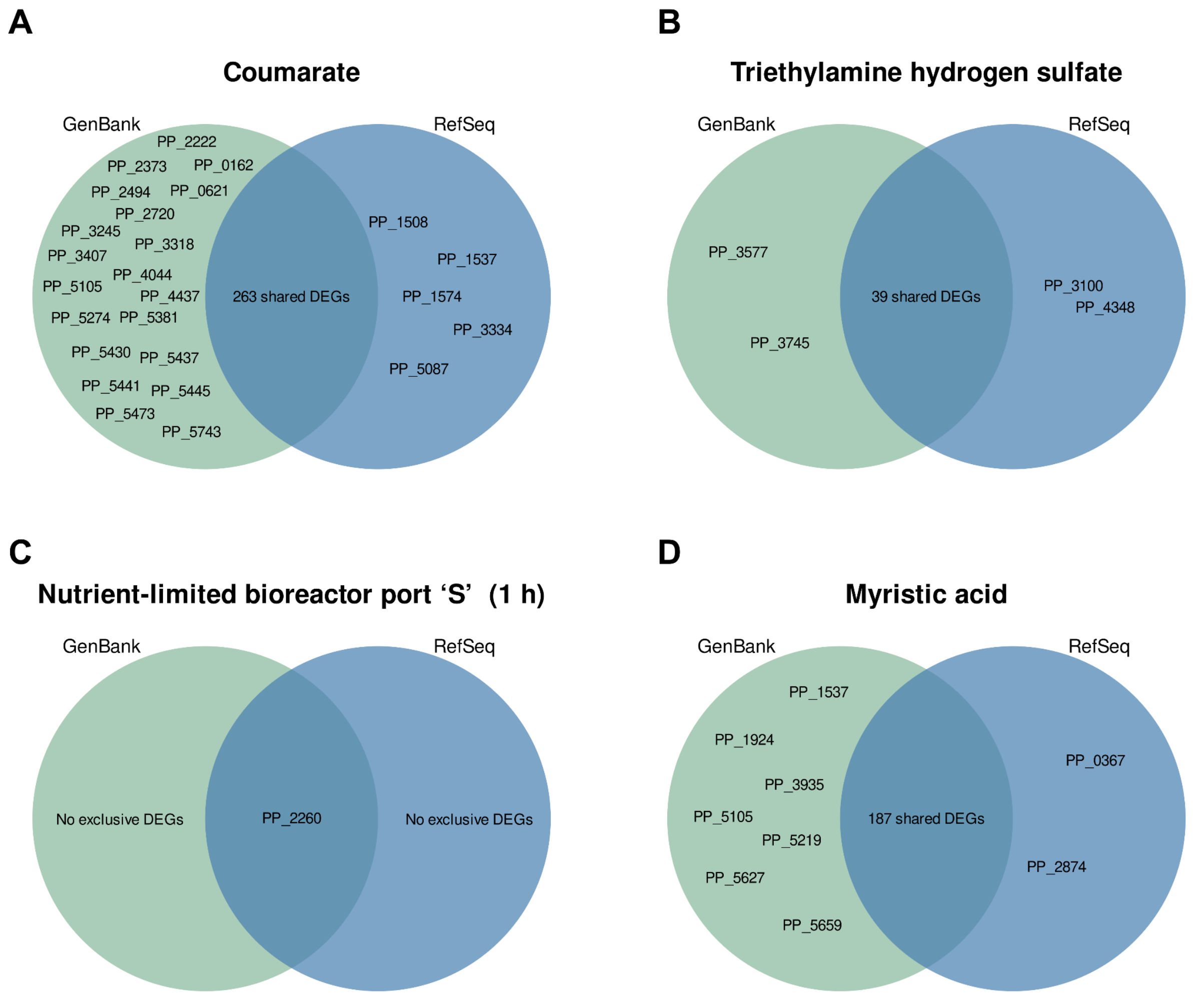

Figure S3. Differences in the output of RNAseq analyses due to the use of different reference transcriptomes. Venn diagrams in panels **A–D** compare the statistically significant differentially expressed genes (DEGs) detected in each RNAseq analysis using GenBank (green) or RefSeq (blue) as reference transcriptomes, considering adjusted *p*-values < 0.05 and absolute $\log_2$ fold change values ≥ 2 as thresholds. Due to the thresholds, the DEGs shown can either be up- or downregulated in the datasets. Data shown here derives from BioProject samples PRJNA796354 (19), PRJNA630234 (28), PRJNA533248 (29), and PRJNA480068 (unpublished) (**Table S1**). The panels in this figure match those of **Figure 4** in the main text.
