# Supplementary material for: Differences in GenBank and RefSeq annotations may affect genomics data interpretation for Pseudomonas putida KT2440
Source: mSphere. 2025 Oct 2;10(10):e00391-25. doi: 10.1128/msphere.00391-25 (PMC12570471; doi:10.1128/msphere.00391-25)
Supplement: Supplemental file legends — Legends for Files S1 to S3. [file msphere.00391-25-s0005.pdf]

Manuscript ID: mSphere00391-25

Differences in GenBank and RefSeq annotations may affect genomics data interpretation for *Pseudomonas putida* KT2440

Supplemental file 1: List of publications compiled from Google Scholar search results using the RefSeq and GenBank *P. putida* KT2440 genome annotation accession codes "NC_002947" and "AE015451", respectively, as search terms.

Supplemental file 2: Comparison of the genomic coordinates in GenBank and RefSeq annotations for the 897 shifted genes identified in this study.

Supplemental file 3: List of *P. putida* KT2440 ORF pairs with overlapping coordinates found exclusively in either RefSeq or GenBank annotations.
